# Real‐Space Observation of Ligand Hole State in Cubic Perovskite SrFeO_3_

**DOI:** 10.1002/advs.202302839

**Published:** 2023-08-18

**Authors:** Shunsuke Kitou, Masaki Gen, Yuiga Nakamura, Kunihisa Sugimoto, Yusuke Tokunaga, Shintaro Ishiwata, and Taka‐hisa Arima

**Affiliations:** ^1^ Department of Advanced Materials Science The University of Tokyo Kashiwa 277‐8561 Japan; ^2^ Center for Emergent Matter Science RIKEN Wako 351‐0198 Japan; ^3^ Japan Synchrotron Radiation Research Institute (JASRI) SPring‐8 Hyogo 679‐5198 Japan; ^4^ Department of Chemistry Kindai University Osaka 577‐8502 Japan; ^5^ Division of Materials Physics Graduate School of Engineering Science Osaka University Toyonaka Osaka 560‐8531 Japan

**Keywords:** electron orbital, ligand hole, orbital hybridization, perovskite oxide, x‐ray diffraction

## Abstract

An anomalously high valence state sometimes shows up in transition‐metal oxide compounds. In such systems, holes tend to occupy mainly the ligand *p* orbitals, giving rise to interesting physical properties such as superconductivity in cuprates and rich magnetic phases in ferrates. However, no one has ever observed the distribution of ligand holes in real space. Here, a successful observation of the spatial distribution of valence electrons in cubic perovskite SrFeO_3_ by high‐energy X‐ray diffraction experiments and precise electron density analysis using a core differential Fourier synthesis method is reported. A real‐space picture of ligand holes formed by the orbital hybridization of Fe 3*d* and O 2*p* is revealed. The anomalous valence state in Fe is attributed to the considerable contribution of the ligand hole, which is related to the metallic nature and the absence of Jahn‐Teller distortions in this system.

## Graphical abstract

Electron density analysis using single‐crystal X‐ray diffraction not only reveals the Fe‐3*d* orbital state in SrFeO_3_ but also provides insights into the distribution of ligand holes formed by Fe‐3*d* and O‐2*p* orbital hybridizations in real space. The absence of Jahn‐Teller distortions in the cubic structure and the metallic nature of this system are strongly associated with the presence of ligand holes.

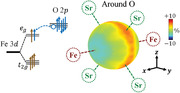

## 1 Introduction

The ionic‐covalent character of the metal‐oxygen bonding in metal oxides has long been discussed. Since the oxygen and metal atomic orbitals mainly contribute to the bonding and antibonding orbitals, respectively, the bonding has a strong ionic nature and hence the formal ionic valence of metal can be well defined in general. Nonetheless, when the formal valence of metal is anomalously high, the contribution of the oxygen 2*p* orbital to the antibonding orbital becomes dominant, presumably resulting in the ligand hole state.^[^ ^1^ ^]^ The ligand holes are considered to play a significant role in the electrical transport^[^ ^2^, ^3^ ^]^ and magnetism^[^ ^4^ ^]^ in cuprates,^[^ ^5^, ^6^ ^]^ nickelates,^[^ ^7^, ^8^ ^]^ cobaltates,^[^ ^9^, ^10^, ^11^ ^]^ and ferrates.^[^ ^11^, ^12^, ^13^, ^14^, ^15^ ^]^

**Figure 1:**
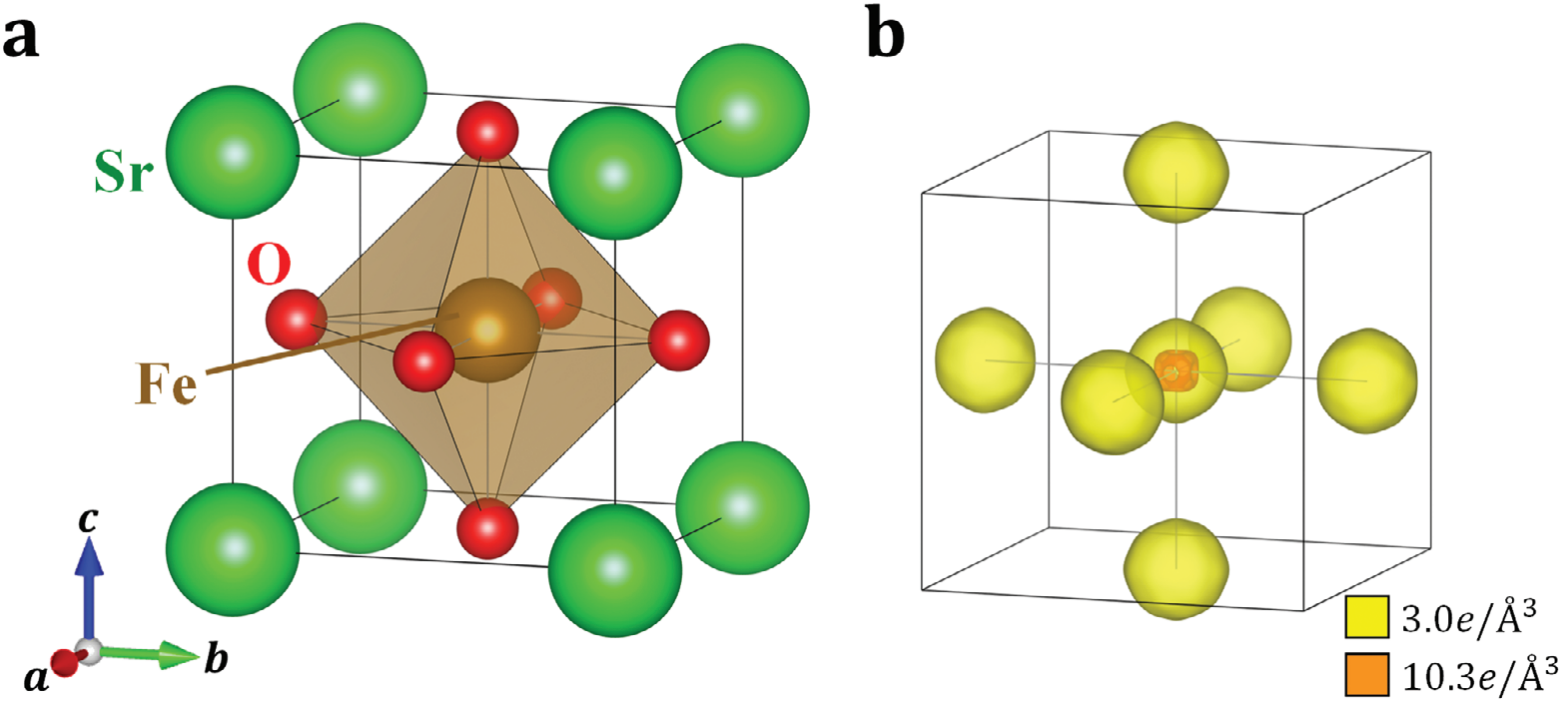
a) Crystal structure and b) valence electron density distribution of SrFeO_3_ at 30 K. Yellow and orange iso‐density surfaces show electron‐density levels of 3.0 and 10.3e/Å^3^, respectively.

SrFeO_3_ is an archetypal tetravalent ferrate compound, in which at formally, four electrons occupy the Fe 3*d* orbitals. It forms the perovskite‐type structure with the cubic space group $Pm\bar{3}m$ (**Figure** **1**a) and exhibits metallic conductivity.^[^ ^16^ ^]^ The local symmetry at the Fe site is $m\bar{3}m$. Each Fe atom is surrounded by six O atoms to form a regular octahedron without Jahn‐Teller distortion, where the 3*d* orbitals are split into the lower‐lying triplet (*t*_2*g*_) and the higher‐lying doublet (*e_g_*). The high‐spin state of 3*d* ^4^ corresponds to the $t_{2g}^{3}e_{g}^{1}$ electron configuration, which causes some anisotropy in the valence electron density. However, previous X‐ray photoelectron spectroscopy and X‐ray absorption spectroscopy measurements suggest that the ground state consists of mixed 3*d* ^4^ and 3*d* ^5^ *L* (*L*: ligand hole) states.^[^ ^11^, ^12^ ^]^ In the extreme limit of the 3*d* ^5^ *L* state, the electron density around the Fe site should be spherical because of the $t_{2g}^{3}e_{g}^{2}$ electron configuration. Theoretically, the ligand hole formed by the 2*p‐*3*d* hybridization is expected to stabilize novel itinerant magnetic phases.^[^ ^17^, ^18^ ^]^ In fact, despite its simple crystal structure, various magnetic phases including a quadruple‐***q*** topological spin structure appear in the magnetic‐field‐versus‐temperature phase diagram in SrFeO_3_.^[^ ^14^, ^15^ ^]^ Furthermore, the crystal structure and physical properties are greatly changed by slight oxygen vacancies,^[^ ^19^, ^20^, ^21^, ^22^, ^23^, ^24^ ^]^ because this system has charge instability of unusual tetravalent iron cations.

Although the ligand holes in the crystal have been observed by spectroscopy measurements,^[^ ^11^, ^12^ ^]^ no one has ever seen where the ligand holes exist in real space. To observe the spatial distribution of the holes, the measurement with high‐wavevector (*Q*) resolution is indispensable. In this study, we observe the valence electron density distribution of SrFeO_3_ by electron density analysis using state‐of‐the‐art synchrotron X‐ray diffraction (XRD). The number of 3*d* electrons is estimated from the anisotropic distribution of the valence electron density around the Fe site. It is also confirmed that the valence electron density around the O site along the Fe―O―Fe axis is slightly reduced.

## 2 Results and Discussion

### 2.1 Crystal Structure of SrFeO_3_

The XRD experiments of SrFeO_3_ detected no structural phase transitions down to 30 K, which is consistent with the previous neutron diffraction experiment.^[^ ^23^ ^]^ As a result of the structural analysis, no signs of oxygen vacancy and no anomaly in the atomic displacement parameters of oxygen were confirmed. Here, the structural parameters were determined with high accuracy by performing a high‐angle analysis utilizing the advantages of high‐energy X‐rays,^[^ ^25^ ^]^ where only reflections with sin *θ*/λ > 0.6Å^‐1^ (d < 0.833Å) were used. The obtained structural parameters of SrFeO_3_ are summarized in Tables S1 and S2 (Supporting Information).

### 2.2 Valence Electron Density Around the Fe Site

Figure 1b shows the valence electron density distribution of SrFeO_3_ at 30 K. No valence electron density larger than 3*e*/Å^3^ is observed around the Sr site, which is consistent with the Sr^2 +^ (5*s* ^0^) state. In contrast, valence electrons are observed around the Fe and O sites, as shown by yellow iso‐density surfaces. An orange iso‐density surface for higher electron distribution is observed only around the Fe site.

**Figure 2:**
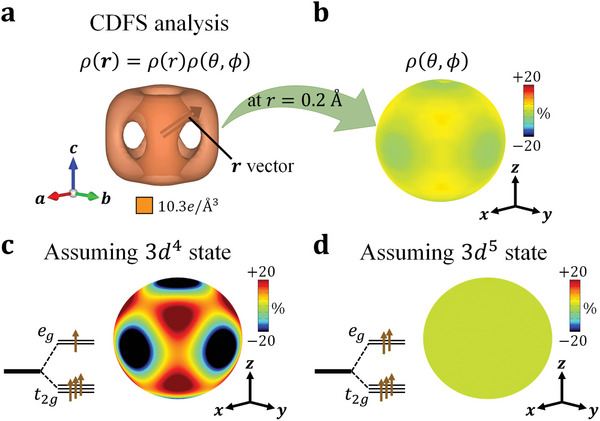
a) Iso‐density surface of valence electrons around the Fe site. b) Color map of the electron density at a distance *r* = 0.2Å from the Fe nucleus. The ***x***‐, ***y***‐, and ***z***‐axes are parallel to the global ***a***‐, ***b***‐, and ***c***‐axes, respectively. Energy diagrams and color maps of the calculated direction dependence of electron density for the c) 3*d* ^4^ and d) 3*d* ^5^ states assuming an isolated Fe atom. The color bar scale is represented by $\frac{\left[\rho \left(\theta ,\phi \right)-N_{e}/4\pi \right]}{N_{e}/4\pi }\times 100\;\left[\%\right]$. *N_e_* is the number of 3*d* electrons.

**Figure 3:**
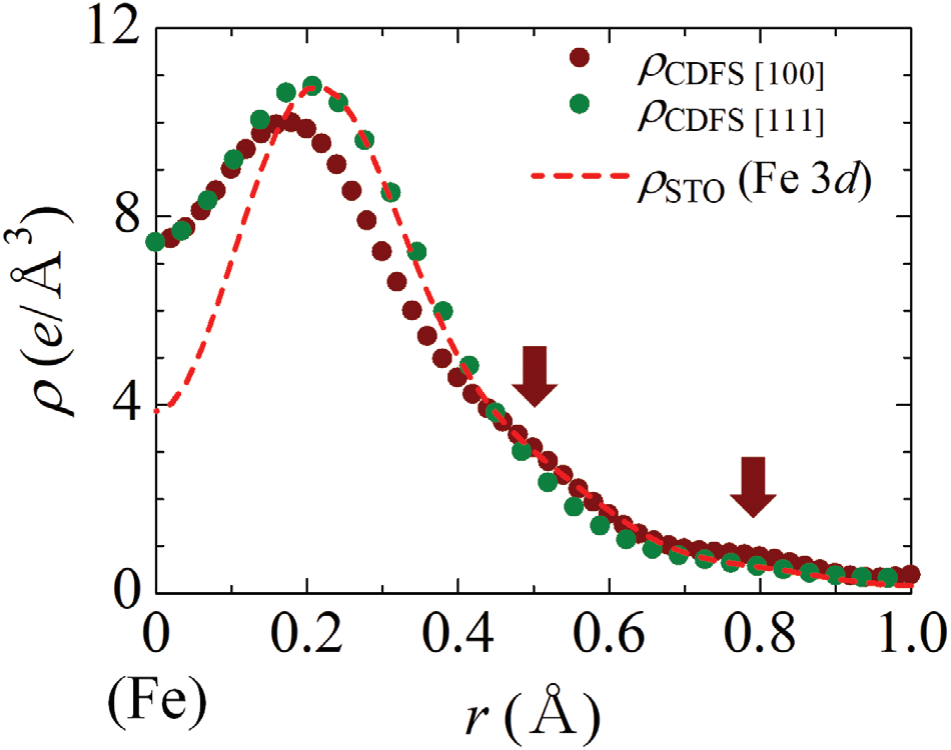
Valence electron densities as a function of the distance *r* from the Fe nucleus. Brown and green dots show the electron densities in the [100] and [111] directions, respectively, obtained by the CDFS analysis. A red broken line shows the 3*d* electron density ρ_STO_ of an isolated Fe ion calculated by the Slater‐type orbital.^[^ ^27^, ^28^, ^29^ ^]^ Detailed methods for calculating the valence electron density are described in Supporting Information. Here, the vertical axis for ρ_STO_ is arbitrarily scaled.

**Figure** **2**a shows the iso‐density surface around the Fe site with the site symmetry $m\bar{3}m$. The shape is clearly distinct from a sphere: there are six hollow holes toward the six ligand oxygens. To quantify the anisotropy of the valence electron density ρ(***r***) around the Fe site, the density at a distance *r* = 0.2Å from the Fe nucleus, which corresponds to the peak top of ρ(*r*) (see **Figure** **3**), is shown by a color map on a sphere (Figure 2b). The maximum and minimum electron densities are present along the <111> and <100> axes, respectively; ρ_max_ = 10.76e/Å^3^ and ρ_min_ = 9.85e/Å^3^. Figure 2c,d show surface color maps of ρ(θ, ϕ) for the calculated electron density considering the high‐spin 3*d* ^4^ and 3*d* ^5^ states for an isolated Fe ion, respectively. To accurately evaluate the anisotropy of the obtained valence electron density distribution of SrFeO_3_, a comprehensive comparison using first‐principles calculations, considering all electrons, is required. However, in this study, we assume the 3*d* ^4^ and 3*d* ^5^ states to discuss the anisotropy of the localized 3*d* electrons around the Fe site. In the case of 3*d* ^4^, we assume that an electron occupies each *e_g_* orbital with a probability of 1/2. Note that ρ(θ, ϕ) for an isolated ion is related just to the spherical harmonics. A clear anisotropy shows up in the 3*d* ^4^ state in contrast to the completely isotropic electron density in the 3*d* ^5^ state. Here, we extract an approximate relation between the number of Fe 3*d* electrons *N_e_* and ρ_min_/ρ_max_; $N_{e}=\;1.773{\left(\frac{\rho_{\min}}{\rho_{\max}}\right)}^{2}+1.163\frac{\rho_{\min}}{\rho_{\max}}+2.096$ (3 ≤ *N_e_* ≤ 5) by considering the experimental resolution (*d* > 0.28 Å) (see Section 2 and Figure S4, Supporting Information). Since the ratio ρ_min_/ρ_max_ obtained by the CDFS analysis is 0.915, the number of Fe 3*d* electrons is estimated to be *N_e_* =  4.64(8), which is consistent with the previous reports of X‐ray absorption spectroscopy measurement (*N_e_* =  4.7)^[^ ^11^ ^]^ and cluster model calculation (*N_e_* =  4.8).^[^ ^26^ ^]^

Radial distributions of the electron density along the [100] and [111] axes around the Fe site are shown in Figure 3. The electron density in the [111] direction toward the nearest Sr atom has a single‐peak structure derived from the 3*d* orbital *r* = 0.2 Å. The electron density of Fe 3*d* calculated by the Slater‐type orbital (STO) of an isolated ion^[^ ^27^, ^28^, ^29^ ^]^ is also plotted as a red broken line, which is in good agreement with the experimental results in the [111] direction. Here, the experimental resolution d > 0.28 Å is considered when performing the inverse Fourier transform (the details are described in Supporting Information). On the other hand, the electron density in the [100] direction toward the ligand O has one clear peak corresponding to the 3*d* orbital with two shoulder‐like structures around *r* = 0.5 and 0.8 Å. The peak top of the electron density from the Fe nucleus is 0.05 Å closer in the [100] than in the [111] direction. These features may arise from the hybridization between the Fe 3*d* and O 2*p* orbitals.

### 2.3 Valence Electron Density Around the O Site

**Figure 4:**
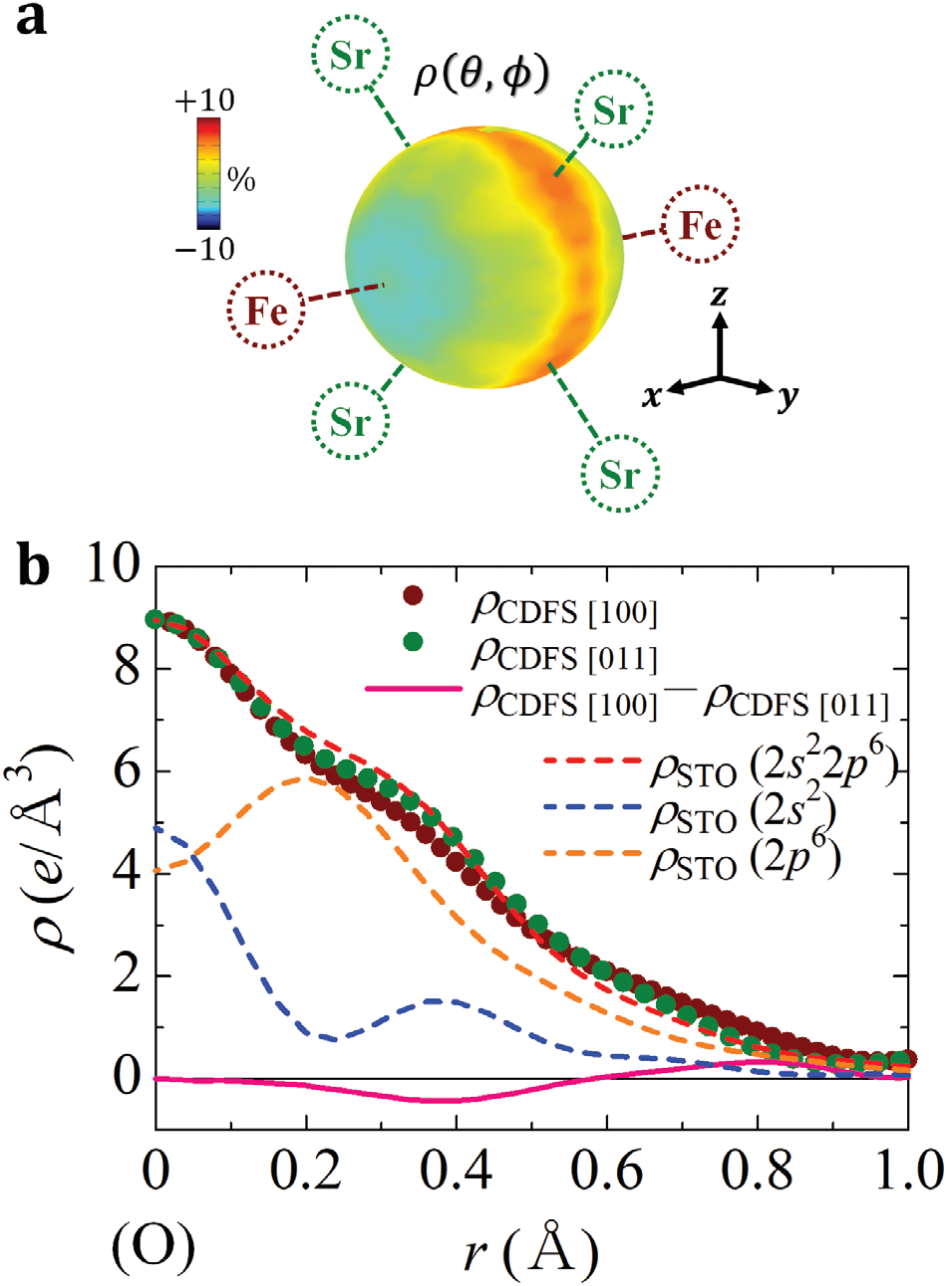
a) Color map of the electron density at a distance *r* = 0.4Å from the O nucleus at (0, 1/2, 1/2). Fe and Sr atoms are present in the [±100] and [0 ±1 ±1] directions, respectively. b) Valence electron densities as a function of the distance *r* from the O nucleus. Brown and green dots show the electron densities in the [100] and [011] directions obtained by the CDFS analysis, respectively. Pink line shows the difference in electron density between the [100] and [011] directions. Blue, orange, and red broken lines show the electron densities ρ_STO_ of oxygen 2*s* ^2^, 2*p* ^6^, and 2*s* ^2^2*p* ^6^ calculated by the Slater‐type orbitals,^[^ ^27^, ^28^, ^29^ ^]^ respectively. Detailed methods for calculating the valence electron density are described in Supporting Information. The vertical value for ρ_STO_ is arbitrarily scaled.

Finally, we focus on the valence electron density around the O site with the site symmetry 4/*mm*.*m*. Since the corresponding valence of Fe obtained by the CDFS analysis was 3.36(8) because of *N_e_* =  4.64(8), the oxygen valence is estimated to be − 1.79(3), which deviates from the ideal closed‐shell value of − 2. That is, the valence electron density distribution around the O site should not be isotropic. **Figure** **4**a shows a color map of ρ(θ, ϕ), which is the electron density at a distance *r*  =  0.40 Å from the O nucleus, obtained from the CDFS analysis. The observed electron density has some anisotropy. The highest electron density exists toward the surrounding four Sr atoms. On the other hand, the lowest electron density is observed in the [100] direction toward Fe. Brown and green dots in Figure 4b show 1D plots of the valence electron density against the distance from the O nucleus in the [100] and [011] directions, respectively. The difference between the two electron‐density profiles is maximum around *r*  =  0.4 Å, as shown in the pink line. We also confirmed that there is no significant anisotropy in the radial direction perpendicular to the [100] direction (Figure S6, Supporting Information). Blue, orange, and red broken lines show the electron densities of oxygen 2*s* ^2^, 2*p* ^6^, and 2*s* ^2^2*p* ^6^ calculated by the STO.^[^ ^27^, ^28^, ^29^ ^]^ The experimentally obtained electron density in the [011] direction (green dots) well agrees with to the electron density of oxygen 2*s* ^2^2*p* ^6^ (red broken line), whereas that in the [100] direction (brown dots) is lower. Furthermore, the difference between the electron densities in the two directions (pink line) mainly corresponds to the contribution of the electron density of oxygen 2*p* ^6^ (orange broken line). These results suggest the existence of ligand holes accommodated in the O 2*p_σ_*―Fe 3*d* antibonding (*σ* ^*^) orbital, which is consistent with previous *p‐d* charge‐transfer cluster‐model calculations^[^ ^12^, ^26^ ^]^ and small holes in O 2*p* densities of states predicted by first‐principles calculations.^[^ ^30^, ^31^ ^]^ Furthermore, while a slight anisotropy in the charge distribution around the Fe site was predicted by the first‐principles calculations,^[^ ^30^ ^]^ the distribution of ligand holes around the O site was captured for the first time by the CDFS analysis.

We observed deviations from ideal Fe^4+^ and O^2−^ states caused by the orbital hybridization as valence electron density distributions. The considerable contribution of the 3*d* ^5^ *L* state seems to be related to the metallic nature^[^ ^16^ ^]^ and absence of the Jahn‐Teller distortion.^[^ ^23^ ^]^ This unusual state, in which various magnetic phases appear in a magnetic‐field,^[^ ^17^, ^18^ ^]^ is easily destroyed by slight charge shifts due to oxygen vacancies, resulting in changes in the crystal structure and the physical property.^[^ ^19^, ^20^, ^21^, ^22^, ^23^, ^24^ ^]^ Thus, the observed *p‐d* hybridization may support the unstable charge state of this system. Our experimental technique will be useful in investigating changes in oxidation state due to oxygen vacancies in this system and negative charge transfer states in other systems such as YBa_2_Cu_3_O_7−δ_^[^ ^6^ ^]^ and SrCoO_3_^[^ ^9^ ^]^ using the electron density analysis in the future.

## 3 Conclusion

The orbital state in SrFeO_3_ has been investigated by synchrotron XRD experiments using a high‐quality single crystal. We have obtained the valence electron density distribution in SrFeO_3_ by the CDFS analysis. The number of Fe 3*d* electrons estimated by the anisotropy is *N_e_* =  4.64(8), which indicates the mixed 3*d* ^4^ and 3*d* ^5^ *L* states. The ligand hole is directly observed around the O site. Our experimental observation of the ligand hole may be a touchstone for future theoretical calculations and offers new possibilities for the study of chemical bonding in transition‐metal compounds.

## 4 Experimental Section

### Single Crystal Growth

Single crystals of SrFeO_3_ were obtained by high‐pressure oxygen annealing of large single crystals of the oxygen‐deficient perovskite SrFeO_2.5_ with brownmillerite‐type structure as described in Refs. [14, 32] A single crystal of SrFeO_2.5_ was grown by a floating‐zone method in an Ar gas flow. The obtained cylindrical crystal with a diameter of ≈4 mm was cut into a suitable size for a gold capsule and then treated with oxidizer NaClO_3_ for 1 h at 873 K and 8 GPa. Tiny SrFeO_3_ crystals were obtained by crashing a part of the cylindrical crystal.

### X‐Ray Diffraction Measurements

XRD experiments were performed on BL02B1 at a synchrotron facility SPring‐8 in Japan.^[^ ^33^ ^]^ The dimensions of the SrFeO_3_ crystal for the XRD experiment were 50 × 30 × 30 µm^3^. A He‐gas‐blowing device was employed to cool the crystal to 30 K. The X‐ray wavelength was λ  =  0.31020 Å. A 2D detector CdTe PILATUS, which had a dynamic range of ≈10^7^, was used to record the diffraction pattern. The intensities of Bragg reflections with the interplane distance *d* > 0.28 Å were collected by CrysAlisPro program^[^ ^34^ ^]^ using a fine slice method, in which the data were obtained by dividing the reciprocal lattice space in increments of Δω  =  0.1°. Intensities of equivalent reflections were averaged, and the structural parameters were refined by using Jana2006.^[^ ^35^ ^]^

### Electron Density Analysis

A core differential Fourier synthesis (CDFS) method was used to extract the valence electron density distribution around each atomic site.^[^ ^25^, ^36^ ^]^ [Kr], [Ar], and [He] type electron configurations were regarded as core electrons for Sr, Fe, and O atoms, respectively. As a result, Sr 5*s*, Fe 3*d*, and O 2*s*/2*p* valence electrons should remain after the subtraction of the core electron density distribution. The detailed process for extracting the valence electron density distribution was described in Ref. [36].

## Conflict of Interest

The authors declare no conflict of interest.

## Supporting information

Supporting Information

## Data Availability

The data that support the findings of this study are available in the supplementary material of this article.
