# Supplementary material for: Real‐Space Observation of Ligand Hole State in Cubic Perovskite SrFeO3
Source: Adv Sci (Weinh). 2023 Aug 18;10(29):2302839. doi: 10.1002/advs.202302839 (PMC10582404; doi:10.1002/advs.202302839)
Supplement: Supplementary file 1 — Supporting Information [file ADVS-10-2302839-s001.pdf]

ADVANCED SCIENCE
Open Access

Supporting Information

for *Adv. Sci.*, DOI 10.1002/advs.202302839

Real-Space Observation of Ligand Hole State in Cubic Perovskite $SrFeO_3$

Shunsuke Kitou, Masaki Gen, Yuiga Nakamura, Kunihisa Sugimoto, Yusuke Tokunaga, Shintaro Ishiwata and and Taka-hisa Arima*

Supporting Information of

Real-Space Observation of Ligand Hole State in Cubic Perovskite $SrFeO_3$

Shunsuke Kitou, Masaki Gen, Yuiga Nakamura, Kunihisa Sugimoto, Yusuke Tokunaga, Shintaro Ishiwata, and Taka-hisa Arima

CONTENTS

1. Crystal structure analysis. 2

2. Electron density analysis. 5

1. Crystal structure analysis.

Figure S1 shows the temperature dependence of the lattice constant of $SrFeO_3$. We observed no signs of structural phase transition such as the appearance of additional peaks or peak splitting. Figure S2 shows the $|F_o|^2$-$|F_c|^2$ plot as a result of the structure analysis of $SrFeO_3$ at 30 K. F_o and F_c correspond to the experimental and calculated crystal structural factors, respectively. The obtained structural parameters of $SrFeO_3$ at 30 K are summarized in Tables S1 and S2. Here, the structural parameters were determined with high accuracy by performing a high-angle analysis, where only reflections with $\sin\theta/\lambda > 0.6$ Å^{-1} ($d < 0.833$ Å) were used for the refinement. When the occupancy of the O site was set as a free parameter, the R factors were not improved and the occupancy of the O site was analyzed to be slightly larger than 1 (Tables S2). This warranted that the sample crystal was not affected by oxygen deficiency.

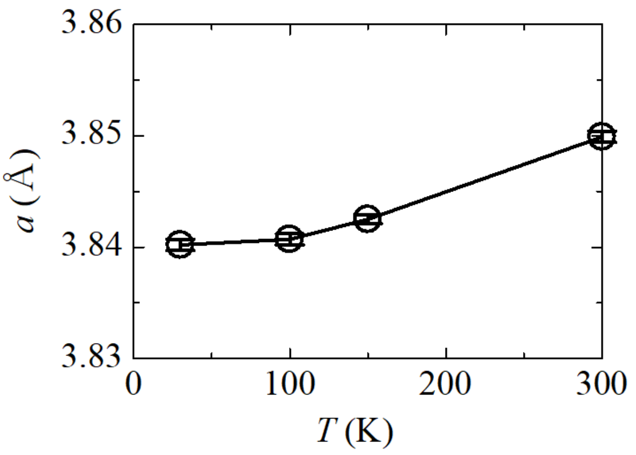

Fig. S1. Temperature dependence of the lattice constant of $SrFeO_3$.

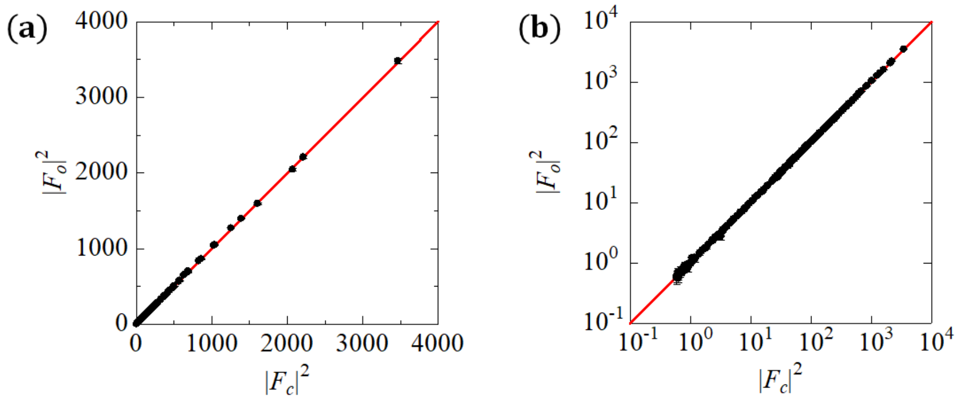

Fig. S2. (a) Linear and (b) logarithmic $|F_o|^2$-$|F_c|^2$ plots as a result of the structure analysis of $SrFeO_3$ at 30 K, respectively.

Table S1. Structural parameters of $SrFeO_3$ at 30 K. The space group is $Pm\bar{3}m$ (No. 221) and $a = 3.8402(5)$ Å. Note that $U_{12} = U_{23} = U_{13} = 0$ for 1*a*, 1*b*, and 3*c* sites.

Atom	Wyckoff position	Site symmetry	*x*	*y*	z	$U_{11}(= U_{22})$ (Å^2)	U_{33} (Å^2)
Sr	1*a*	$m\bar{3}m$	0	0	0	0.002214(10)	$= U_{11}$
Fe	1*b*	$m\bar{3}m$	1/2	1/2	1/2	0.002482(10)	$= U_{11}$
O	3*c*	$4/mm.m$	1/2	1/2	0	0.00522(3)	0.00377(3)

Table S2. Summary of crystallographic data of $SrFeO_3$ at 30 K.

Wavelength (Å)	0.3102 Å
Crystal dimension (μm^3)	50×30×30
Space group	$Pm\bar{3}m$
a (Å)	3.8402(5)
Z	1
F(000)	88
$(\sin\theta/\lambda)_{max}$ (Å^{-1})	1.79
N_{total}	8898
N_{unique} (sinθ/λ>0.6Å^{-1} / all)	303 / 325
Average redundancy	27.378
Completeness (%)	99.09
When the occupancy of oxygen was fixed to one.	
$N_{parameters}$	5 (four *U's* + scale factor)
R_1 (sinθ/λ>0.6Å^{-1} / all)	0.65% / 0.64%
wR_2 (sinθ/λ>0.6Å^{-1} / all)	1.04% / 1.02%
GOF (sinθ/λ>0.6Å^{-1} / all)	0.85 / 0.84
When the occupancy of oxygen was not fixed and only high-angle reflections were used.	
$N_{parameters}$	6
R_1 (sinθ/λ>0.6Å^{-1})	0.65%
wR_2 (sinθ/λ>0.6Å^{-1})	1.03%
GOF (sinθ/λ>0.6Å^{-1})	0.84
Occupancy of the O site	1.009(6)

2. Electron density analysis.

Figures S3a and S3b show one-dimensional plots of the electron density of Fe $3d^5$ and O $2s^2 2p^6$ calculated by the Slater-type orbital (STO) of the isolated atom, respectively [1]. $\rho_{\mathrm{STO}}(d > 0\ \text{Å})$ is the raw data of the calculated electron density. $\rho_{\mathrm{STO}}(d > 0.28\ \text{Å})$ is the electron density considering the same resolution as the experiment, which was calculated by the following processes.

(i) The crystal structure factor $F(\boldsymbol{K})$ was calculated by the Fourier transform of $\rho_{\mathrm{STO}}(d > 0\ \text{Å})$ as

$$F(\boldsymbol{K}) = \int_{\text{unit cell}} \rho_{\mathrm{STO}}(d > 0\ \text{Å}) e^{-i\boldsymbol{K}\cdot\boldsymbol{r}} d\boldsymbol{r}. \tag{S1}$$

(ii) $\rho_{\mathrm{STO}}(d > 0.28\ \text{Å})$ was calculated by the inverse Fourier transform of the calculated $F(\boldsymbol{K})$ with $d_{\min} = 0.28\ \text{Å}$ using

$$\rho_{\mathrm{STO}}(d > 0.28\ \text{Å}) = \frac{1}{V} \sum_{|\boldsymbol{K}| \le |\boldsymbol{K}|_{\max}} F(\boldsymbol{K}) e^{i\boldsymbol{K}\cdot\boldsymbol{r}}. \tag{S2}$$

Here, $|\boldsymbol{K}|_{\max} = 2\pi/d_{\min}$. Although the distributions of $\rho_{\mathrm{STO}}(d > 0\ \text{Å})$ and $\rho_{\mathrm{STO}}(d > 0.28\ \text{Å})$ roughly match each other, the truncation effects of the inverse Fourier transform can be seen around $r = 0\ \text{Å}$ in Figures S3a and S3b, and around $r = 0.2\ \text{Å}$ in Figure S3b.

Figure S4 shows the relationship between the number of Fe $3d$ electrons N_e and the calculated $\rho_{\min}/\rho_{\max}$ described in Figure 2. If it were not for the truncation effects, $\rho_{\min}/\rho_{\max}$ would be linear to N_e, as shown by the black line. By considering the experimental resolution $d > 0.28$ Å, N_e is approximately related with $\rho_{\min}/\rho_{\max}$ as

$$N_e = 1.773 \left(\frac{\rho_{\min}}{\rho_{\max}}\right)^2 + 1.163 \frac{\rho_{\min}}{\rho_{\max}} + 2.096. \tag{S3}$$

Figures S5(a) and S5(b) show the valence electron densities as a function of the distance r from the O nucleus obtained by the CDFS analysis using reflection with $d > 0.28$ Å and $d > 0.35$ Å, respectively. A negative peak corresponding to the ligand hole is clearly observed around $r = 0.4$ Å (pink line) even in the data with slightly reduced experimental resolution ($d > 0.35$ Å), which indicates that the ligand hole distribution is intrinsic.

We also investigated the valence electron density profile in the [010] direction (Fig. S6), which has a similar radial distribution in the [011] direction shown by the green dots in Fig. 4(b).

Figure S7 shows the temperature dependence of the electron densities in $SrFeO_3$. No clear change with temperature confirms that the anisotropy of the electron density around the Fe and O sites is intrinsic.

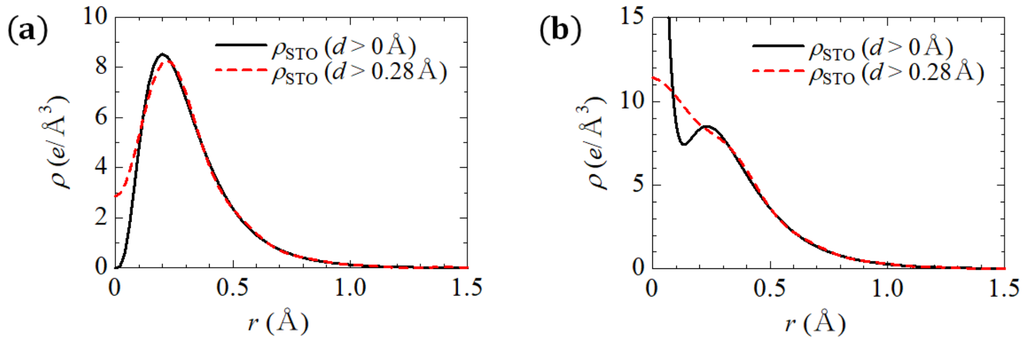

Fig. S3. Truncation effect on the calculated electron densities of (a) Fe $3d^5$ and (b) O $2s^2 2p^6$. Black solid and red broken lines show the electron densities calculated using $F(\boldsymbol{K})$ with $d > 0$ Å and $d > 0.28$ Å, respectively.

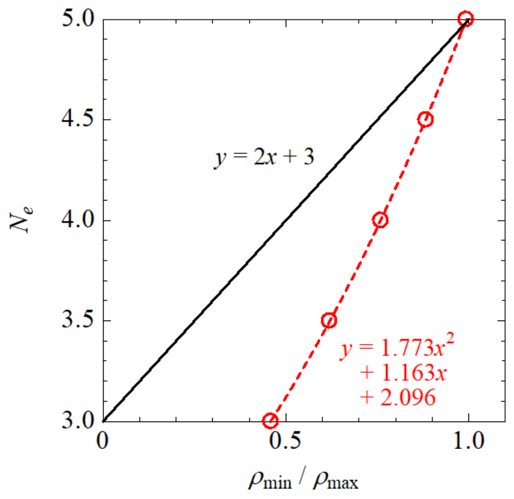

Fig. S4. The relationship between the number of 3*d* electrons N_e and the calculated $\rho_{\mathrm{min}}/\rho_{\mathrm{max}}$, which is described in the main manuscript. Black and red lines show the relation calculated using $F(\boldsymbol{K})$ with $\rho_{\mathrm{STO}}(d > 0\ \text{Å})$ and $\rho_{\mathrm{STO}}(d > 0.28\ \text{Å})$, respectively.

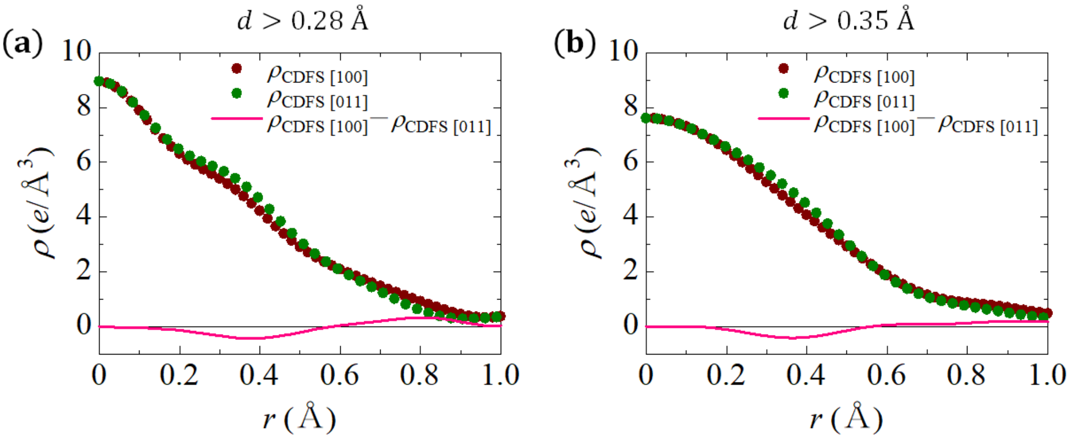

Fig. S5. Experimental resolution dependence of the valence electron densities as a function of the distance r from the O nucleus at 30 K. (a) and (b) are obtained by the CDFS analysis using reflection with $d > 0.28$ Å and $d > 0.35$ Å, respectively.

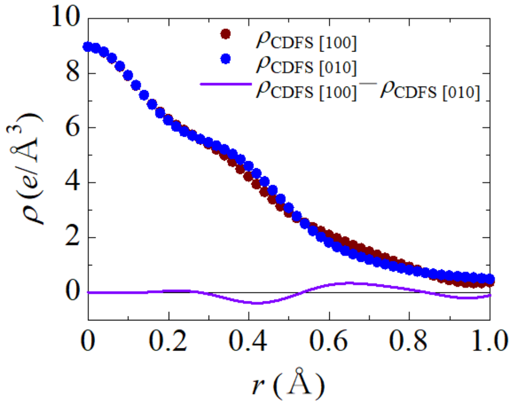

Fig. S6. Valence electron densities as a function of the distance r from the O nucleus. Brown and blue dots show the electron densities in the [100] and [010] directions obtained by the CDFS analysis, respectively. Purple line shows the difference in electron density between the [100] and [010] directions.

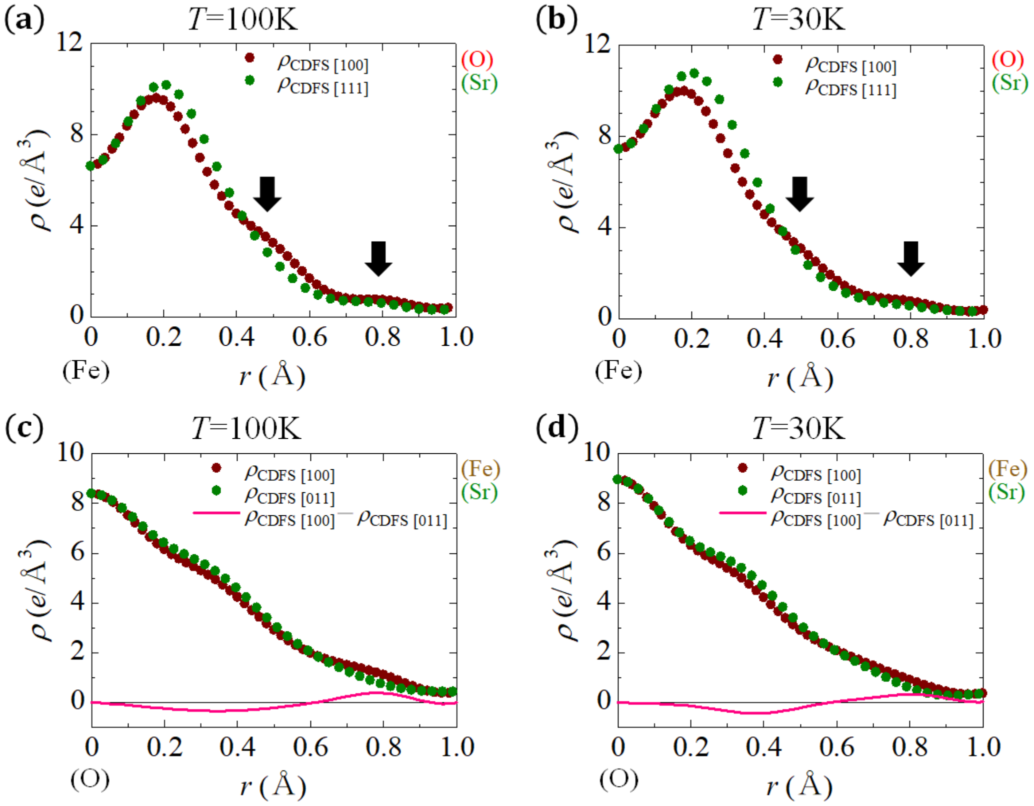

Fig. S7. One-dimensional profiles of the valence electron densities along several high-symmetry axes as a function of the distance r from the Fe nucleus at (a) 100 K and (b) 30 K and those from the O nucleus at (c) 100 K and (d) 30 K. The pink lines in (c) and (d) show the difference in electron density between the [100] and [011] directions.

References.

1. Z. Su, Coppens, P. Relativistic X-ray Elastic Scattering Factors for Neutral Atoms Z = 1-54 from Multiconfiguration Dirac-Fock Wavefunctions in the 0-12 $Å^{-1}$ sin θ/λ Range, and Six-Gaussian Analytical Expressions in the 0-6 $Å^{-1}$ Range. *Acta Crystallogr.* **1997**, *A53*, 749-762; P. Macchi, P. Coppens, Relativistic analytical wave functions and scattering factors for neutral atoms beyond Kr and for all chemically important ions up to I^-. *Acta Crystallogr.* **2001**, *A57*, 656-662.
